# Kaposi’s Sarcoma-Associated Herpesvirus, the Etiological Agent of All Epidemiological Forms of Kaposi’s Sarcoma

**DOI:** 10.3390/cancers13246208

**Published:** 2021-12-09

**Authors:** Aude Jary, Marianne Veyri, Adélie Gothland, Valentin Leducq, Vincent Calvez, Anne-Geneviève Marcelin

**Affiliations:** 1 Service de Virologie, Hôpital Pitié-Salpêtrière, AP-HP, Institut Pierre Louis d’Épidémiologie et de Santé Publique (iPLESP), INSERM, Sorbonne Université, 75013 Paris, France; adelie.gothland-ext@aphp.fr (A.G.); valentin.leducq@sorbonne-universite.fr (V.L.); vincent.calvez@aphp.fr (V.C.); anne-genevieve.marcelin@aphp.fr (A.-G.M.); 2 Service d’Oncologie Médicale, Hôpitaux Universitaires Pitié Salpêtrière-Charles Foix, AP-HP, Institut Pierre Louis d’Épidémiologie et de Santé Publique (iPLESP), INSERM, Sorbonne Université, 75013 Paris, France; marianne.veyri@aphp.fr

**Keywords:** KSHV, HHV-8, oncogenic virus, Kaposi’s sarcoma, latency, oncogenic viral proteins, LANA-1

## Abstract

**Simple Summary:**

Kaposi’s sarcoma-associated herpesvirus (KSHV) is one of the seven oncogenic viruses currently recognized by the International Agency for Research on Cancer. Its presence for Kaposi’s sarcoma development is essential and knowledge on the oncogenic process has increased since its discovery in 1994. However, some uncertainties remain to be clarified, in particular on the exact routes of transmission and disparities in KSHV seroprevalence and the prevalence of Kaposi’s sarcoma worldwide. Here, we summarized the current data on the KSHV viral particle’s structure, its genome, the replication, its seroprevalence, the viral diversity and the lytic and latent oncogenesis proteins involved in Kaposi’s sarcoma. Lastly, we reported the environmental, immunological and viral factors possibly associated with KSHV transmission that could also play a role in the development of Kaposi’s sarcoma.

**Abstract:**

Kaposi’s sarcoma-associated herpesvirus (KSHV), also called human herpesvirus 8 (HHV-8), is an oncogenic virus belonging to the *Herpesviridae* family. The viral particle is composed of a double-stranded DNA harboring 90 open reading frames, incorporated in an icosahedral capsid and enveloped. The viral cycle is divided in the following two states: a short lytic phase, and a latency phase that leads to a persistent infection in target cells and the expression of a small number of genes, including LANA-1, v-FLIP and v-cyclin. The seroprevalence and risk factors of infection differ around the world, and saliva seems to play a major role in viral transmission. KSHV is found in all epidemiological forms of Kaposi’s sarcoma including classic, endemic, iatrogenic, epidemic and non-epidemic forms. In a Kaposi’s sarcoma lesion, KSHV is mainly in a latent state; however, a small proportion of viral particles (<5%) are in a replicative state and are reported to be potentially involved in the proliferation of neighboring cells, suggesting they have crucial roles in the process of tumorigenesis. KSHV encodes oncogenic proteins (LANA-1, v-FLIP, v-cyclin, v-GPCR, v-IL6, v-CCL, v-MIP, v-IRF, etc.) that can modulate cellular pathways in order to induce the characteristics found in all cancer, including the inhibition of apoptosis, cells’ proliferation stimulation, angiogenesis, inflammation and immune escape, and, therefore, are involved in the development of Kaposi’s sarcoma.

## 1. Introduction

Kaposi’s sarcoma-associated herpesvirus (KSHV), also called human herpesvirus 8 (HHV-8), is an oncogenic virus that was discovered in 1994 by Chang et al. in the USA [1]. They described, for the first time, two viral fragments of 330 and 631 base pairs (bp) in skin biopsies issued from AIDS patients with Kaposi’s sarcoma (KS). These fragments, identified through a representational difference analysis assay, were phylogenetically closed to the Epstein–Barr virus (EBV), and to the saimiri herpesvirus (*Saimirine herpesvirus* 2, SaHV-2). The following year, KSHV was also detected in two hemopathies, the primary effusion lymphoma (PEL) [2], a rare type of non-Hodgkin’s malignant lymphoma (LMNH) and a lymphoproliferative syndrome, Multicentric Castleman’s disease (MCD) [3]. Thus far, epidemiological and molecular studies have subsequently confirmed the association between KSHV and Kaposi’s sarcoma, and it became one of the ten carcinogenic infectious agents in humans listed by the International Agency for Research on Cancer (IARC) (https://monographs.iarc.who.int/list-of-classifications, accessed on 4 December 2021.)

## 2. Classification

On the basis of phylogenic analysis, KSHV belongs to the *Herpesviridae* family and the *Gammaherpevirinae* subfamily, and, to date, it is the first and only human *Rhadinovirus* identified [4,5]. KSHV is also related to the rhadinoviruses *herpesvirus saimiri*, identified in 1968 in squirrel monkeys [6] and *herpesvirus ateles* found in spider monkeys in 1972 [7]. Since, several studies have described a rhadinovirus related to KSHV infecting macaques, African green monkeys, monkeys and chimpanzees [8,9,10,11,12].

## 3. Structure

### 3.1. Viral Particle

**Figure 1:**
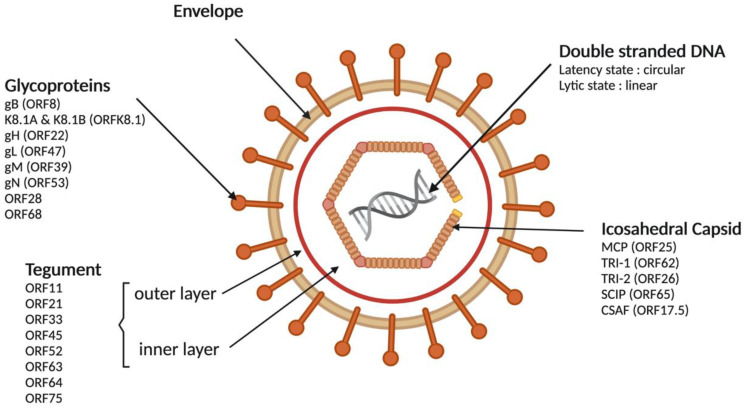
Structure of the Kaposi’s sarcoma-associated herpesvirus viral particle. Similar to the other human herpesviruses, the KSHV virion is composed of the following four morphologically distinct components: The double-stranded viral DNA genome, an icosahedral capsid that encloses the viral DNA, a lipid envelope derived from cellular membranes and the electron-dense material between the capsid and the envelope, which is defined as tegument. In each layer, several viral proteins are incorporated. CSAF: Scaffolding or assembly protein; DNA acid desoxyribonucleic; gX: Glycoprotein; MCP: Major capsid protein; ORF: Open reading frame; SCIP: Small capsomer-interacting protein; TRI 1 and 2: Triplex component 1 and 2. Created with BioRender.com.

As with all herpesvirus, KSHV is a large double-stranded DNA virus of approximately 165 to 170 kilo bp associated with a typical herpesvirus icosahedral capsid composed of four structural proteins (MCP (major capsid protein), TRI-1, TRI-2 (triplex component 1 and 2), SCIP (small capsomer-interacting protein) and CSAF (scaffolding or assembly protein)) [13,14] that is not a part of the capsid but is essential for its assembly. The capsid is surrounded by a tegument, defined as an electron dense material, and composed of an inner and external layers associated with the following eight proteins encoded by the ORF: 11, 21, 33, 45, 52, 63, 64 and 75 [15,16]. They may contribute to the early events of viral replication as well as the entry of the genome during the primary infection. Finally, the tegument is surrounded by an envelope deriving from the nucleus membrane (lipid bilayer) of a KSHV-infected cell [17]. Eight glycoproteins are incorporated in this layer (gB, K8.1A, K8.1B, gH, gL, gM, gN) and involved in the interaction with host cells [14,15,18,19,20,21]. KSHV’s viral particle size is about 110 to 150 nm in diameter (Figure 1).

### 3.2. Viral Genome

**Figure 2:**
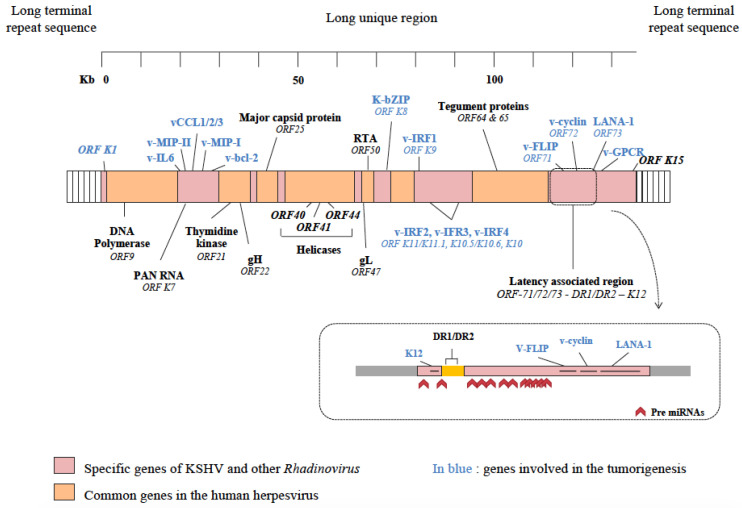
Structure of the Kaposi’s sarcoma herpesvirus genome. The long central unique region, about 137 kilo bp in length, is flanked with highly GC-rich 801-pb-long terminal repeat sequences. The genome harbors 90 open reading frames (ORFs) and 13 pre microRNAs (miRNAs) encoding for 25 miRNAs. Most of the ORFs are common in the human herpesvirus (region in orange), whereas fifteen are specific to the Rhadinovirus (K1 to K15, region in red). As oncovirus, KSHV also encoded proteins involved in tumorigenesis (in blue) and having proliferative, antiapoptotic, angiogenic, immuno-evasion and inflammatory properties. The main genes encoding proteins involved in the latency state, including LANA-1, v-FLIP, v-cyclin, mtRNAs and Kaposin, are grouped in the latency-associated region located at the right end of the genome. DNA acid desoxyribonucleic; gX: Glycoprotein; Kb: Kilo base; K-bZIP: K basic leucine zipper protein; LANA-1: latency associated nuclear antigen 1; MCP: Major capsid protein; ORF: Open reading frame; PAN RNA: Polyadenylated nuclear RNA; RTA: Replication and transcription activator; TRI 1 and 2: Triplex component 1 and 2; v-IL6: Viral interleukin 6; v-MIP: Viral macrophage inflammatory protein; v-CCL: Viral-encoded chemokines; v-IRF: Viral interferon regulatory factor; v-cyclin: Viral cyclin; v-FLIP: Viral Fas-associated protein with death domain-like interleukin-1β-converting enzyme/caspase-8-inhibitory protein; v-GPCR: Viral G-protein coupled receptor.

KSHV’s genome was firstly sequenced from the PEL cell line, BC-1 [5]. The central long unique region (LUR), about 137 kilo bp in length, is flanked with highly GC-rich 801 pb long terminal repeat sequences [5]. The LUR includes the 90 open reading frames (ORFs) and the 13 pre microRNA (miRNA) encoding for 25 miRNAs. Most of the ORFs are common in the human herpesvirus (from ORF4 to ORF75) [14], whereas fifteen are specific to the *Rhadinovirus* (K1 to K15) (Figure 2). Several of these ORFs encode numerous proteins involved in lytic and latent infection programs. The viral genes encoded by KSHV also include cellular homologous genes that may be included in the common or specific genes (for example, v-IL-6 (viral interleukin 6), v-BCL-2, v-FLIP (viral Fas-associated protein with death domain-like interleukin-1β-converting enzyme/caspase-8-inhibitory protein), v-Cyclin) [22]. Furthermore, consistent with its transforming potential, KSHV encodes numerous proteins with proliferative, antiapoptotic, angiogenic, immuno-evasion properties and inflammation [23]. More recently, KSHV has been found to generate circular RNAs (circRNAs) from several KSHV genes, most abundantly from K10 (vIRF4, viral interferon regulatory factor 4), K7.3 and polyadenylated nuclear (PAN) RNA. All the KSHV circRNAs are incorporated into KSHV virions and are potentially expressed as immediate early products in newly infected cells [24].

During the latency state, the KSHV genome appears in a circular form, called an episome (or circular mini chromosome), not integrated in the genome of the host cell. In the lytic phase, the KSHV genome takes a linear conformation in order to replicate and express the various proteins inherent in this phase.

## 4. Viral Replication

As with all herpesviruses, KSHV undergoes either latent or lytic infection programs that are differentiated by complex but characteristic genes expression patterns: latent infection, which is its default pathway, and episodes of lytic reactivation, leading to the production of infectious particles and the death of the host cell.

### 4.1. Entry in the Cell

The entry of KSHV is a sequential, multistep process [25]. First, the surface glycoproteins of KSHV bind nonspecifically onto its target cell. Multiple interactions with cell surface proteoglycans facilitate its attachment, and these are primarily mediated by gB, gHgL and K8.1A [20,26,27]. Although these proteoglycans are not essentials, they enhance the entry of KSHV by concentrating the virions on the surface of the target cell [20,26,28]. The viral glycoproteins can then interact with their specific cellular receptors, such as the heparan sulfate, the α3β1 integrin, the xCT (cystine-glutamate transporter) or DC-SIGN (Dendritic Cell-Specific Intercellular adhesion molecule-3-Grabbing Non-integrin), depending on the target cell, and stimulate different endocytosis pathways (mostly fusion or endocytosis) leading to the entry of the viral particle [29,30,31,32]. Once the nucleocapsid is in the cytosol, KSHV undergoes activated intracellular transport by the cytoskeletal machinery to perinuclear regions, where it delivers the viral genome into the nuclei, resulting in the expression of viral genes and the reprogramming of host cell genes [32,33,34].

### 4.2. KSHV Latent Cycle

In the latent state, the viral circular episome appears attached to the chromosome of the host cell and replicates during each cell division according to the cellular replication machinery to be distributed in each daughter cell. This is a non-productive replication that leads neither to the production of infectious virions nor the lysis of the host cell. Only a small fraction of viral genes is expressed, allowing the KSHV to escape the immune system and to maintain the genome in dividing cells, resulting in a persistent latent infection.

The central protein of the latent phase is the LANA-1(latency-associated nuclear antigen 1) protein encoded by the ORF-73 [35]. LANA-1 is involved in the maintenance of the viral genome in the nucleus by interacting with the histones of the host DNA via MeCP2 (methyl CpG binding protein 2) and DEK, leading to the transmission of the KSHV genome to daughter cells during cell division. LANA-1 also has the role of transcriptional regulator, in particular by repressing the expression of the RTA (replication and transcription activator) protein that is responsible for the entry of the virus into the multiplication phase. The other two main proteins expressed are two homologues of human cellular proteins as follows: the v-cyclin encoded by the ORF-72 and the v-FLIP encoded by the ORF-71/K13. These three latency proteins will also be able to modulate various oncogenic processes, such as cell proliferation, differentiation and survival, allowing the transformation and immortalization of the cell, and thus induce the various phenotypic characteristics that are observed in KSHV-associated diseases [15].

A few other proteins are also expressed during the latent cycle, including the Kaposin proteins, encoded by ORF-K12, and 25 miRNAs (micro RNAs), non-coding single-stranded RNAs of approximately 19–23 nucleotides, encoded by 13 pre-miRNAs [36,37,38]. The latter are involved both in the regulation of the viral cycle—for example, miRNA-K7-5p and miRNA-K9-5p repress the expression of the viral protein RTA at the post-transcriptional level [39]—and also in the regulation of the cell cycle and in the interaction with the host cell to promote the persistence of the virus and the development of related diseases [37,38,40,41,42].

### 4.3. KSHV Lytic Cycle

**Figure 3:**
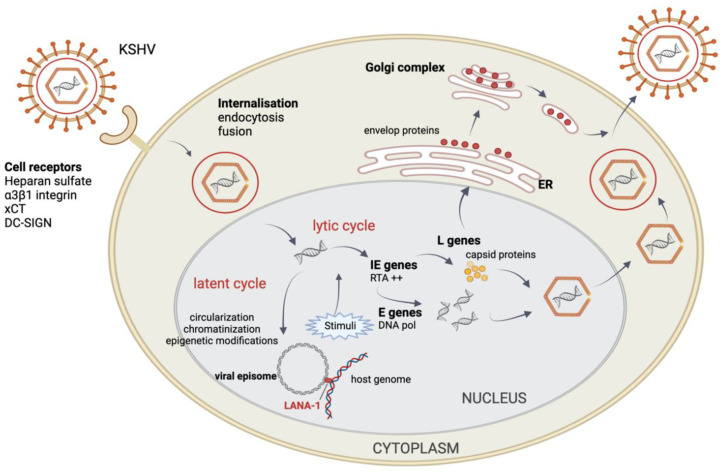
Viral cycles of Kaposi’s sarcoma-associated herpesvirus. The viral glycoproteins of KSHV interact with their specific cellular receptors (different receptors available depending on the target cell) and stimulate different endocytosis pathways leading to the entry of the viral particle. Once the nucleocapsid is in the cytosol, it is transported to the cell nucleus where only the viral genome is released. In the nucleus, the following two viral cycles can be achieved: (i) the lytic cycle leading to the production of new infectious particles and host cell lysis, (ii) the latent cycle leading to the persistence of the viral episome in the nucleus, binding through LANA-1 to the host cell genome. Diverse stimuli (physiological, chemical, etc.) lead to the reactivation of the KSHV and the expression of lytic genes following a temporal and sequential expression pattern divided into the following three phases: immediate early (IE), early (E) and late (L). xCT: cystine-glutamate transporter; DC-SIGN: Dendritic Cell-Specific Intercellular adhesion molecule-3-Grabbing Non-integrin. Created with BioRender.com.

KSHV is able to reactivate and enter in the replicative phase under the influence of various protein stimuli (ex: PKC, ERK) and physiological (ex: hypoxia, oxidative stress, reactive oxygen species, etc.) [43,44] or chemical substances such as TPA (tetradecanoyl phorbol myristyl acetate) and n-butyrate [45]. This replicative phase leads to the lysis of the infected cells and the production of new infectious viral particles. Similar to the other herpesviruses, KSHV expresses genes following a temporal and sequential expression pattern divided in the following three phases: immediate–early (IE), early (E) and late (L). The IE genes encode transactivators of the E and L genes, including, in particular, the ORF-50 encoding the RTA protein, the leader for the initiation of the lytic phase [46,47]. To do so, the RTA protein associates with the cellular transcription factor RBP-Jk in order to activate its own promoter and, thus, maintains itself at a sufficient level for perpetuating the lytic cycle [48,49]. Other genes, such as ORF K8 and ORF57, are also expressed concomitantly with ORF50. More recently, PAN RNA, a non-coding viral RNA encoded by ORF-K7, has also been reported to play a role in viral reactivation by sequestering the LANA-1 protein, and thus lifting its inhibition on lytic gene expression [50,51]. After the IE phase, the E genes expressed an encoded viral protein primarily required for DNA replication and gene expression, in particular, among others, the viral DNA polymerase (ORF9) involved in the replication of the viral genome, the viral thymidine kinase (ORF21) and the viral phosphotransferase (ORF36, homologue of the UL97 protein kinase encoded by human cytomegalovirus), bZIP, vIRF-1 (viral interferon regulatory factor), v-IL-6, v-CCLs (viral-encoded chemokines) and v-GPCR (viral G-protein coupled receptor) [52]. Finally, about 24 h after the initiation of the lytic phase, the L genes encoding structural and maturation proteins of the viral particles are expressed. Once all of the three phases have been achieved, the assembly of the new viral particle begins in the nucleus. The KSHV genome is incorporated into the newly synthesized capsids in the nucleus, then acquires teguments in the cytoplasm and, buds through host membranes to obtain envelopes. Finally, the viral progeny are released from the host cell [48] (Figure 3).

Importantly, the boundary between the latency and lytic phases is not obvious as it was supposed. Thus far, the idea that there are interactions between the two phases of the cycle, and that the expressions of some viral transcripts overlap on both phases, is well integrated now [53,54,55,56].

## 5. Epidemiology

### 5.1. KSHV Seroprevalence and Transmission

Unlike other human herpesviruses, KSHV’s seroprevalence differs according to the geographical area worldwide with a North–South gradient. Indeed, in Sub-Saharan Africa, more than 50% of the population is infected with KSHV, whereas in Western Europe, North America and Asia, KSHV infection remains anecdotic, with less than 10% of the population having encountered this virus. Around the Mediterranean basin and in Eastern Europe, KSHV seroprevalence varies between 10 and 30%, depending on the studies [22,55] (Table 1). The reasons for these disparities are not yet clear. However, it appears that environmental factors such as infectious agents (malaria and co-infection with other parasites) [57,58,59] or the presence of soils rich in metals (such as aluminum, silica or iron) in some parts of the world may increase the risk of KSHV transmission as well as the risk of Kaposi’s sarcoma development [60,61,62,63].

#### 5.1.1. Transmission in Countries with Low Seroprevalence

In non-endemic regions, KSHV transmission mainly occurs in men who have sex with men (MSM), with a seroprevalence ranging from 30 to 60% depending on the HIV status [64,65,66,67,68]. Recently, Liu et al. reported an overall seroprevalence of 33% among MSM whatever their HIV status [69]. Several studies have shown that KSHV transmission is linked to risky sexual behavior, including the number of different sexual partners [70], the duration of sexual activity [71], HIV infection [71,72], history of other sexually transmitted infections (STIs) [73], the use of oral or inhaled poppers and oral–penile [64,74] or oral–anal contacts [75]. Although saliva appears to play an essential role in the transmission, it remains uncertain. Indeed, in MSM infected with KSHV, KSHV-DNA is frequently and intermittently detected in saliva [76], whereas in seminal fluid, its detection varies depending on the HIV status and the studied population [77,78,79,80]. On the other hand, KSHV is rarely found in a urine or anal site [81]. One of the hypotheses on transmission in this population is based on the practice of others forms of intimate contact, especially during oral–anal sex [65,82]. This hypothesis is also reinforced by the fact that in the heterosexual population, no evidence of sexual transmission appears, although the results of different studies are inconsistent [73,83,84,85,86]. The two other possible ways of transmitting KSHV in these regions are blood and transplantation. However, transmission through blood is not clearly proven with a seroprevalence described in drug addicts, hemophiliacs and poly transfused individuals comparable to that of blood donors [87,88,89,90,91]. The safety of blood products by systematic leucocytes depletion probably contributes to this low risk of transmission. On the contrary, KSHV transmission by solid organ transplantation should be considered [92]. According to the studies, seroconversion occurs in 14 to 31% of cases, regardless of the type of graft [93,94,95].

#### 5.1.2. Transmission in Endemic Regions

In highly endemic countries, the epidemiology and modes of KSHV transmission appear to be different with a seroprevalence that is often already high in childhood and increases with age until a plateau is reached toward the end of puberty [96,97]. Several studies have shown family cases of KSHV seropositivity, suggesting transmission in childhood from mother to child or between siblings [98,99]. Saliva is also thought to play a preponderant role in the transmission [100], and the following hypotheses have been made: pre-chewing of food, application of maternal saliva to arthropod bites or sharing toothbrushes [101,102]. Maternal-to-fetal, perinatal and human milk transmission remains rare [103]. In some parts of Sub-Saharan Africa, Kaposi’s sarcoma is very common due to the importance of co-infection with HIV-1. However, the association between KSHV seropositivity and HIV infection, especially in heterosexual populations, remains uncertain [104,105]. Finally, the risk of transmission by blood transfusion was reported in the literature and should be considered [106].

### 5.2. Molecular Epidemiology

Initially, the study of the KSHV genetic diversity relied on the sequencing of the two genes firstly discovered and led to the identification of three viral subtypes, A, B and C, from lesions of AIDS patients with Kaposi’s sarcoma. However, the low variability of these genes between different subtypes (less than 3%) limited their interest in describing the genetic diversity of KSHV.

#### 5.2.1. ORF-K1

From the end of the 1990s, several teams were interested in ORF-K1, a gene located at the end left of the KSHV genome [107,108], similarly to the genes encoding LMP-1 (latent membrane protein) in EBV and STP (saimiri transformation protein) in SaHV. This ORF encodes for a glycosylated transmembrane protein of 289 amino acids, a structure also found in the immunoglobulin receptor family. Its extracellular domain contains conserved regions (C1 and C2) and two hypervariable regions (VR1 and VR2), whereas the C-terminal intracytoplasmic domain carries a conserved ITAM motif involved in the activation of transduction pathways and the oncogenic process [108,109,110,111]. The variability of this gene, mainly located in the VR1 (54 to 93 AA) and the VR2 (191 to 228 AA) domains, led to the identification of the seven subtypes (A, B, C, D, E, F and Z) currently described with up to 44% of amino acid sequence variability between them. The subtypes B and D differ from subtypes A and C by about 30 and 24%, respectively, whereas subtypes A and C differ by about 15% from each other. These subtypes were also subdivided into genotypic variants (e.g., A1, A2, A3) when their amino acid sequences varied by approximately 10% [108].

**Figure 4:**
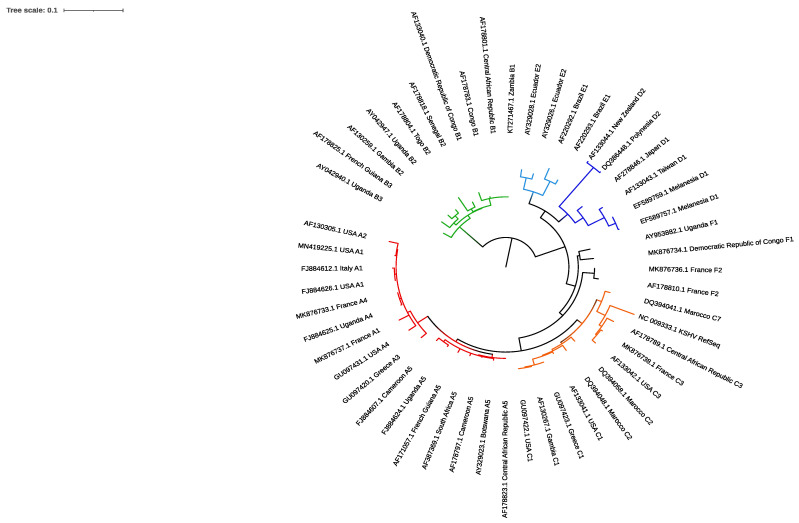
Phylogenetic tree constructed with 54 nucleotides sequences of ORF-K1 and issued from the Genbank database to describe the diversity of Kaposi’s sarcoma-associated herpesvirus worldwide. Phylogenic analysis was performed with PhyML (version 3.0) with the GTR model, four rate categories of gamma shape parameter and 1000 bootstrap resampling. Tree was visualized and managed with the iTOL v6. (Interactive Tree of Life) online tool. Tree is midpoint rooted and branches of each subtype are identified with different colors as follows: subtype A: red, subtype B: green, subtype C: orange, subtype D: dark blue, subtype E: light blue, subtype F: black. Label names are constructed as follows: NCBI identification number, country of origin, subtype/variant.

For now, the diversity of KSHV is mainly related to the origin of the patients; subtypes A and C are found worldwide and particularly in North America, Western Europe, the Mediterranean basin and Asia [112,113,114]. The variant A5, on the contrary, was first, and is mainly, reported in Africa, as is the subtype B [115,116]. The subtype D is described in the Pacific Island and Taiwan [117]; the subtype E is in Native Americans in Brazil [118,119]; the subtype F is in a few individuals in Uganda (variant F1) [120] and, more recently, in Caucasian MSM living in France (variant F2) [121]; finally, the subtype Z is described in a small cohort of children in Zambia [122] (Figure 4).

#### 5.2.2. Other Genes

ORF-K15, located at the right end of the LUR, is the second most variable gene in KSHV and on the basis of a second molecular classification. This gene contains eight alternately spliced exons, and when all eight are included, the transcript encodes a 45 kilo Dalton K15 protein with 12 membrane domains [123]. At least three diverging alleles of K15, with less than 33% amino acid sequence similarity, have currently been identified and were designated as P (predominant), M (minor) and N [124,125]. Other ORFs have also been used to describe KSHV diversity, such as the ORF-26 with nine identified subtypes (A, B, C, D, E, J, K, Q and R) whose geographical distribution is parallel to that of the subtypes obtained with ORF-K1 [126], or even ORF-73. More recently, next generation sequencing (NGS) has made it possible to increase the sequencing capacities and to understand the entire KSHV genome. By whole genome sequencing, four new genes were identified as varying between the following different KSHV strains: K4.2, K8.1, K11/vIRF2 and K12/Kaposin, suggesting that the entire genome should be considered to precisely characterize each strain [127]. In addition, Sallah et al. reported that large-scale genome sequencing is also necessary to capture the full extent of genetic diversity, including KSHV recombination, and provided evidence to suggest a revision of KSHV genotype nomenclature [128].

#### 5.2.3. Molecular Diversity and Pathogenicity

Some studies have suggested that different subtypes of KSHV could have different pathogenic and tumor properties, and thus could be associated with different clinical presentation severities and progressions [129,130,131]. However, the results reported in the literature are inconsistent. For example, White et al. did not describe any difference in the clinical presentation between subtypes A and B in patients with the epidemic KS form in Zimbabwe [132], as Kadyrova et al., who compared patients with classic, post-transplant and epidemic forms in Russia, did [133]. On the other hand, Mancuso et al. described the classic form of KS evolving faster when they involved a subtype A and associated it with higher blood KSHV viral loads [130]. In the past year, we also reported that the KSHV viral load in the blood compartment was higher for subtype A than for subtype C, regardless of the immunovirological status, in MSM with the epidemic KS form [121]. According to Isaac et al., variant A5 in the epidemic KS form in Africa was reported to be associated with more than 10 KS lesion at diagnosis, whereas variants A1 and A4 were reported to be associated with a lower risk of an extensive form and variant A1 with a lower risk of lower limb involvement [115]. In addition, Tozetto et al. reported that subtype B was associated with a better prognosis in patients with epidemic KS in Brazil [134] and Barete et al. reported that subtypes A and B’ have more aggressive forms of KS after transplantation than subtype C [135]. However, most of the studies have been conducted with a small number of cases and all the subtypes were not necessarily represented in the geographic zones where the studies were performed.

On the other hand, a study has shown in vitro that the K1 gene in the epidemic form was associated with a higher transforming activity compared with those in the classic form. This higher activity was also correlated with the severity of the clinical presentation [136].

Thus far, the interest of KSHV typing for the management of KS and for predicting its evolution remains to be defined.

## 6. KSHV Oncogenesis and Kaposi’s Sarcoma

### 6.1. KSHV, Cells Tropism and Risk Factors

After primo-infection, KSHV primarily infects B-cells and endothelial cells in vivo but also can infect several kinds of cells, including dendritic cells, macrophages, epithelial cells, fibroblast and mesenchymal stem cells in laboratory cell culture [25,137]. Then, KSHV enters in a latent state, notably in B lymphocytes and monocytes. Several studies have mentioned the potential involvement of some environmental factors, such as the consumption of chemical molecules from plants [138], the presence of a malaria parasite [139,140] or the absorption of iron found in volcanic soils [63,141], to promote the transmission and the pathogenesis associated with KSHV by stimulating KSHV reactivation.

More classically, iatrogenic immunosuppression after transplantation or HIV infection are strong cofactors promoting KHSV pathologies [142,143]. The postulate of a synergy between HIV and KSHV promoting cell transformation has been put forward, but in-depth studies are still needed. Indeed, some secretory proteins of HIV, such as Tat and Nef, could be released into the bloodstream in order to promote the appearance of Kaposi’s sarcoma [144,145]. In particular, it has been shown in vitro that the Tat protein induces a reactivation of KSHV in order to promote the growth of endothelial cells infected with KSHV in cooperation with the protein K1 [146,147,148,149,150]. The persistence of a detectable HIV-1 RNA viral load in vivo, despite antiretroviral treatment and an effective reconstitution of the immune system, has also been reported as a greater risk factor for developing Kaposi’s sarcoma [151].

Importantly, KSHV is found in the lesions of all epidemiological forms of Kaposi’s sarcoma [152] with an average of one to two copies of the viral genome per infected cell [153], and its presence and persistence has been shown experimentally to be necessary to initiate and maintain tumor growth [154,155]. The modulation of several cellular pathways by the oncogenic viral proteins allows the KSHV-infected cell to override the tumor suppressor and apoptotic signals, leading to its proliferation and immortalization, but also to induce other characteristics found in all types of cancer, including angiogenesis, inflammation and escape from the immune system [23,156]. In Kaposi’s sarcoma, the spindle cells (typical cells infected with KSHV) presumably derive from the endothelial cell line; however, the lymphatic or vascular nature of these cells is still subject to debate. Indeed, the spindle cells express markers of both vascular and lymphatic endothelial cells (VEGF-3, LYVE-1 and podoplanin or CD34, CD31 and CD36, respectively) and possess the phenotypic characteristics of the two cells [157,158,159]. On the other hand, their gene expression profile does not accurately represent either of these two endothelial lineages [160]. In the vast majority of Kaposi’s sarcoma cells, the KSHV virus is found in a persistent state of latency, indicating the central role of viral latent proteins in the development of this disease. However, a small proportion of spindle cells undergoes spontaneous lytic viral reactivation, resulting in the expression of lytic proteins and the production of virions, (<5% according to the literature [161,162]) potentially involved in the proliferation of neighboring cells, by stimulating the production of secreted pro-inflammatory and pro-angiogenic factors, suggesting their crucial roles in the process of tumorigenesis [161,163,164,165].

### 6.2. KSHV Latency Proteins and Oncogenesis

Gene-encoded latency proteins strongly contribute to the establishment of a latent infection and the oncogenesis process. These proteins possess the ability to constitutively and/or transiently modulate cellular signaling pathways that are essential for tumor transformation and the survival of the infected cells, such as PI3K-AKT-mTOR, MAPK and NF-kB [110,166,167] (Table 2). They also participate in the production of pro-angiogenic and pro-inflammatory signals involved in the pathogenesis of cancers associated with KSHV. Among others, LANA-1 is the central protein for the establishment of KSHV latency and is strongly involved in promoting tumorigenesis. First, in the initial establishment of the latent state after primo-infection, LANA-1 recruits many components of the host epigenetic machinery to promote the formation of latent KSHV episomes [168,169]. Moreover, LANA-1 is essential for episomal maintenance, replication and segregation during cell division [35,169]. LANA can interfere with cell cycle progression and apoptosis, in particular by inhibiting the transcription factor p53, and also plays an important role in the activation of the cell proliferation and transformation pathways [22]. LANA-1 also contributes to angiogenesis [170] and participates in cell immortalization by increasing the expression of the telomerase and, thus, prolonging the life of the infected cell [171]. Considering the few other genes expressed, the v-cyclin, v-FLIP, Kaposin and miRNAs are also able to regulate several host-signaling pathways. In particular, KSHV-encoded miRNAs are involved in the maintenance of viral latency and play roles in tumorigenesis by inhibiting the expression of multiple viral and host genes. Some of them intervene in the inhibition of apoptosis, whereas others promote the immune escape by regulating the host response [37,38,40]. Some miRNAs are also able to modulate angiogenesis, cell migration and adhesion or endothelial cells’ transformation, which are essential for the dissemination of KSHV infection and its viral pathogenicity [41,42,172]. Otherwise, for example, v-FLIP can activate a key cellular pathway, the NF-kB pathway, leading to cell survival and proliferation during latency [173], but also inducing the production of inflammatory cytokines and chemokines [174,175]. KSHV also encodes a viral homolog to cellular cyclin, v-cyclin, that associates with cellular cyclin-dependent kinase 6 (CDK6). The v-cyclin-CDK6 complex ensures the phosphorylation and inactivation of cell cycle inhibitor proteins such as p21 and p27, leading to an accelerated transition of the G1/S phase of the cell cycle [176].

### 6.3. Lytic Proteins and Oncogenesis

While latency proteins are expressed in all infected cells, lytic proteins are produced by only a small proportion of tumor cells. However, their presence is essential for the tumorigenesis of KSHV [186]. Indeed, they lead not only to maintenance of the viral infection by producing new infectious particles, but also to the escape of the immune system and the production of cytokine and growth factors. Thus, they can influence surrounding cells by establishing an inflammatory and angiogenic environment favorable to tumor progression. Among them, the main lytic proteins involved in the pathogenicity of the KSHV are, in particular, ORF-K1, v-IL-6, v-BCL2, v-GPCR, v-IRFs 1-4, vCCCL-1, -2 and -3 and v-IAPs, and the majority are homologs of cellular proteins (Table 2).

For example, the K1 protein increases cell survival by activating the anti-apoptotic pathway PI3K-AKT whereas the v-GPCR contributes to angiogenesis by stimulating the secretion of pro-inflammatory and pro-angiogenic factors, such as VEGF, IL-6 and -8, leading to tumor progression in a paracrine fashion [22,110,187,188,189,190]. On the other hand, v-IL-6 is able to activate the JAK-STAT, MAPK-ERK and PI3-AKT pathways through gp130 and might induce cell survival and proliferation [22,55,189]. Viral IL-6 can also induce the excretion of pro-inflammatory cytokines, such as human IL-6, contributing to the pro-inflammatory and pro-angiogenic environment of KS [189]. On the other hand, the v-CCL proteins are involved in the immune escape and the stimulation of angiogenesis in Kaposi’s sarcoma by inducing VEGF [191]. These viral chemokines also have the ability to act as paracrine contributors on the survival of latently infected or uninfected cells, thus participating in KSHV pathogenesis [192]. Finally, the v-IRF proteins are lytic proteins that are able to inhibit the production and signaling of type I interferons (INFs I) by targeting cellular IRFs and leading to a reduction in immune defense [52,193].

## 7. Rationale and Feasibility of KSHV Vaccine

Currently, there is no KSHV vaccine available to prevent KSHV infection or treat its associated diseases. However, Kaposi’s sarcoma remains one of the most common cancers in Africa and still occurs in HIV-1-infected patients, although immunovirological control of the HIV-1 infection has been achieved [194,195]. In addition, the therapeutic approaches to treat Kaposi’s sarcoma are limited, as some are associated with a limit toxic dose [196] and are not readily accessible in resource-limited countries, especially in Sub-Saharan Africa. Thus, the development of a KSHV vaccine could have a major impact on public health worldwide, and more specifically, in endemic areas and on those people who are at risk of KSHV infection or immunosuppressed.

An immune response against KSHV is essential to prevent the development of Kaposi’s sarcoma, and includes the following different immune pathways: (i) Natural killer cells by downregulating the expression of MHC type 1 molecules on tumor cells infected with KSHV [197,198], (ii) KSHV-specific CD8 T-cells’ responses, which is lower in HIV-infected patients with Kaposi’s sarcoma compared with those infected with KSHV but without symptoms [199,200]. KSHV-specific CD8 T-cells were reported to target both early and late lytic proteins, as well as two KSHV proteins, LANA and K12/Kaposin [201]; (iii) neutralizing antibodies (nAbs) are also induced by KSHV infection [202,203] and may represent the most promising way for the development of KSHV prophylactic vaccines. Recently, Mortazavi et al. characterized the antigenic targets of KSHV-specific nAbs and found that, of the eight envelope glycoproteins, the gH/gL complex is the predominant antigenic determinant of KSHV-specific nAbs [204].

## 8. Conclusions

KSHV is one of the seven oncogenic viruses currently known, and it is found in all the epidemiological forms of Kaposi’s sarcoma. However, the mechanisms of transmission and oncogenesis leading to Kaposi’s sarcoma are complex. Indeed, the global distribution of KSHV infection diverges by region, suggesting the impact of cofactors not identified yet. Otherwise, although KSHV encodes several oncogenes that could potentially induce a tumor phenotype, KSHV infection in the general population rarely leads to Kaposi’s sarcoma, suggesting the importance of cofactors, such as immune deficiency. Thus far, further studies are required to improve our knowledge on KSHV’s transmission, molecular characteristics and oncogenesis.

## Figures and Tables

**Table 1:** Seroprevalence of Kaposi’s sarcoma-associated herpesvirus and risk factors of transmission.

	Low	Intermediate	High
World region	North America Western Europe Eastern Asia	Mediterranean basin Eastern Europe Southern America Western Africa	Central Africa Eastern Africa
KSHV Seroprevalence	<10%	10–30%	>50%
Transmission	Sexual Iatrogenic	Sexual Iatrogenic Nonsexual	Childhood Sexual
Risk factors	Risky sexual behavior: STIs including HIV, number of different sexual partners Use of poppers?	Infectious agents (malaria)? Soils rich in metals? Chemical substances from plants?	Infectious agents (malaria)? Soils rich in metals? Chemical substances from plants? Transfusion
At-risk population	MSM Organ transplant patients	MSM Organ transplant patients Elderly men	Children Elderly men Low socio-economic level

MSM: Men having sex with men; STIs: Sexually transmitted infections; HIV: Human immunodeficiency virus; KSHV: Kaposi’s sarcoma-associated herpesvirus.

**Table 2:** Essential gene-encoded proteins involved in latency/lytic cycles and tumorigenesis of Kaposi’s sarcoma-associated herpesvirus.

Proteins or RNA	Gene	Viral Cycle	Cellular Homologs	Essential Functions
LANA-1	ORF73	Latent	-	Inhibition of lytic cycle by inhibiting the expression of RTA [177] Sequestration of KSHV episome in nucleus and transmission to daughters’ cell during mitosis Inhibition of apoptosis by interacting with p53 and pRB Recruitment of DNA methyltransferase [178] Recruitment of host PRC and KAP1 to suppress lytic gene expression [179,180] Cytoplasmic isoform of LANA-1 inhibits cGAS to promote KSHV reactivation [181]
v-cyclin	ORF72	Latent	Cyclin D2	Cell proliferation: v-cyclin-CDK6 => inhibition of p21 and p27 Cell transformation
v-FLIP	ORF71	Latent	FLICE	Inhibition of apoptosis Activation of NF-kB pathway by interaction with NEMO [182] Inhibition of RBP-Jk (co-activator of RTA)
miRNA miRNA-K7-5p miRNA-K9-5p miRNA K1	- - - miR-K11	Latent	-	Immunomodulation, immune escape, inhibition of apoptosis Post transcriptional inhibition of RTA expression Post transcriptional inhibition of RTA expression Activation of NF-kB pathway
Kaposin	ORF-K12	Latent	-	Cell transformation Activation of p18/MK2 pathways
LANA-2 /v-IRF3	ORF 10.5	Latent	-	Inhibition of p53 pathway Immune escape
RTA	ORF50	Lytic	-	Activation of lytic cycle Stimulation of human IL-6 production Inhibition of p53 Activation of LANA-1 expression [177]
K-bZIP /RAP	ORF K8	Lytic	Zta (ZEBRA) in EBV	Bind to RTA protein and suppression of its transactivation Interaction with CREB binding protein Stimulation of p53 and p21 and promotion of cell cycle arrest
v-IL-6	ORF K2	Lytic	IL-6	Inhibition of apoptosis Interaction with cellular cycle Activation of JAK-STAT, MAPK-ERK and PI3-AKT pathways => Cell survival, and pro-inflammatory and pro-angiogenic environment Functional modulation of B cell by promoting CSR [183]
v-GPCR	ORF 74	Lytic	IL8 receptor, CXCR2	Interaction with MAPKs, PI3K-AKT and NF-kB pathways Cell transformation Stimulation of angiogenesis
v-bcl-2	ORF 16	Lytic	Bcl-2	Inhibition of apoptosis
K1 (KIST)	ORF K1	Lytic	STP in SaHV	Inhibition of NF-kB pathway Cell survival by activating PI3K-AKT pathway Immune escape by the ITAM motif
v-IRF1	ORF K9	Lytic	IRF1	Inhibition of NF-kB pathway Cell proliferation Inhibition of genes expression induced by INF
K14	ORF K14	Lytic	-	Inhibition of NF-kB pathway
PAN RNA	ORF K7	Lytic	-	LANA-1 sequestration [50] Recruitment of histone demethylase to the viral chromosome [184] Generated circRNA potentially involved in early infection [24]
v-MIP-1 v-MIP-2 v-MIP-3	ORF K6 ORF K4 ORF K4.1	Lytic	MIP-1alpha - MIP-1béta	Stimulation of angiogenesis Inhibition of naïve and active human NK cells [185]

LANA: Latency Associated Nuclear Antigen; ORF: Open Reading Frame; FLIP: Flice-Inhibitory Protein; cGAS: cGMP-AMP synthase; PRC: polycomb repressive complex; STP: Saimiri Transforming Protein; IL-6: Interleukin 6; CSR: class-switch recombination; pRB: Retinoblastoma Protein; RTA: Replication and Transcription Activator; PAN ARN: polyadenylated nuclear Acid ribonucleic; IRF: Interféron Regulatory Factor; RBP-Jk: Recombination Signal Binding Protein For Immunoglobulin Kappa J Region; HVS: herpèsvirus samiri; vGPCR: viral G-protein coupled receptor; VEGF: vascular endothelial growth factor; EBV: Epstein–Barr Virus; ITAM: immunoreceptor tyrosine-based activation motifs; v-MIP: viral macrophage inflammatory protein; CDK6: cellular cyclin-dependent kinase 6; NK: natural killer.
